# Supplementary material for: Overexpression of ApHIPP26 from the Hyperaccumulator Arabis paniculata Confers Enhanced Cadmium Tolerance and Accumulation to Arabidopsis thaliana
Source: Int J Mol Sci. 2023 Oct 10;24(20):15052. doi: 10.3390/ijms242015052 (PMC10606507; doi:10.3390/ijms242015052)
Supplement: Supplementary file 1 [file ijms-24-15052-s001.zip › ijms-2632121-supplementary.pdf]

Table S1 Primers used in experiment

Gene name	Forward primer	Reverse primer
pBI121-ApHIPP26	ATGGGTGTTCTTGATCATG	CATGACCACACAAGCCGCT
pROKII-ApHIPP26	ATGGGTGTTCTTGATCATG	CATGACCACACAAGCCGCT
ApACTIN	ATAAGCATCCTTTTGCCCCAT	TCCTTTGTTTCCTTCTCTCG
AtACTIN	CTTAACCCAAAGGCCAACAGA	GCAAGGTCAAGACGGAGGAT
qApHIPP26	GTCGTTGGAGCCTAAATTCC	CTTCTTGTCGTAGACTCCGG
qAtNAS3	TATCCGATCGTTGAGCCGTG	CATTTTCGCGTGGGTCTTGG
qAtHMA2	GCTGAGGATTGCGTGGTT	GATAAGTCCACAAGCACAAGCAC
qAtNRAMP3	CCGAGTCCAAGAAGCGCTAA	CCGCGTTAACCAAACCGATG
qAtNRAMP6	TTTGCAATTGCGTTGCTTGC	CTCCAGCTGAGCCACCTATC
qAtYSL2	TCGTTCGAGAGAGCATTCCG	TTAAGCCAGCTCCATAGGCG
qAtCAX3	GACTTGTGGAAACGCGACAG	ACACAACAGGCCCATAAGCA
qAtZIP4	CATCGGTTTATCCCTGGGGG	TGCAGTTCCAATCCCGATCC
qAtIRT3	AATCCGGCCTCTAATCGCAG	CGATCCCTATCGGGGTTGTG
qAtARF11	CGGATTCGATCTCACGAGCA	TGCACCTTGATTCGGCTTCT
qAtIAA27	GGGTGTTCTCCGACGCTATT	CGGAACAGCCGGTTTAGTGA
qAtSAUR1	GGTACCAAAAGGGTGTTTGGC	CAACGTGACAAGGGATCGTG
qAtSNRK2	TCGAGCGAGGCCAAAAGATT	ACCTTGCCTCGTCTTCACTG
qAtHAB1	CCCACCGCGGAAGTTCTTAT	GACACGGCAAAAGCATCCTC
qAtHAI1	TTGTGGCATTGCCTGATCCT	ACAGCCATGGCCATCGTAAA
qAtERF1	CTAATCGAGCAGTCCACGCA	GTCCCGAGCCAAACCCTAAT
qAtETR2	GCTCCTTCGGTTTCGACGTA	TACGTGTAACTGCCCGGTTC

Table S2 DEGs involved in the plant hormone biosynthesis and the plant hormone signal transduction

KEGG	Gene ID	Gene name	CdWT_1	CdWT_2	CdWT_3	CdOE5_1	CdOE5_2	CdOE5_3	log2FC
AUX/IAA	AT4G29080	IAA27	6.71	3.18	4.4	22.75	13.37	5.86	1.465269049
	AT5G25890	IAA28	20.67	16.51	25.56	6.6	5.97	2.91	-2.091713308
ARF	AT1G19850	MP	11.76	18.61	15.04	2.79	0.91	1.83	-3.144910658
	AT2G46530	ARF11	4.55	5.41	3.66	14.42	11.04	15.05	1.481647722
GH3	AT4G37390	BRU6	18.5	18.81	28.94	3.76	0.89	8.8	-2.311459542
	AT5G13320	PBS3	89.7	117.06	159.2	22.08	23.2	18.17	-2.594847411
	AT5G54510	DFL1	8.99	8.76	10.95	2.63	2	0.67	-2.516826016
	AT4G27260	WES1	6.21	28.67	13.63	4.52	4.2	6.81	-1.930577835
	AT1G28130	GH3.17	12.7	17	17.71	0.46	0.43	0.33	-5.393976158
	AT5G13370	GH3.15	62.24	68.96	74.49	8.4	12.7	21.41	-2.26108895
	AT2G23170	GH3.3	28.29	59.45	52.96	7.18	3.44	7.18	-3.030276078
SAUR	AT4G00880	SAUR31	30.92	21.34	21.57	200.28	155.7	92.35	2.517537028
	AT4G13790	SAUR25	12.64	4.63	13.15	0	0.27	0	-6.72665579
	AT4G34750	SAUR49	4.8	2.19	5.34	10.7	19.53	8.56	1.646716416
	AT5G18080	SAUR24	1.58	0.46	0.35	9.06	11.02	6.32	3.374475122
	AT2G45210	SAUR36	14.05	17.71	27.8	2.48	6.3	4.62	-2.155605672
	AT4G12410	SAUR35	2.4	1.65	1.64	0.69	0	0.36	-3.708666759
	AT5G50760	SAUR55	16.34	20.54	28.58	0.8	0.75	3.39	-3.708666759
	AT2G28085	SAUR42	15.66	25.76	15.63	3.03	0	0.3	-4.38122619
	AT5G18030	SAUR21	0.75	0.46	0.61	3.01	4.91	1.64	2.338830349
	AT5G53590		48.11	60.12	71.71	26.1	19.9	13.97	-1.664567699
PP2C	AT1G72770	HAB1	72.57	98.24	122.82	26.95	34.11	28.19	-1.737763869
	AT5G51760	AHG1	0.2	0.43	1.38	0	0	0.04	-5.502489668
	AT5G57050	ABI2	43.84	37.02	49.82	16.85	15.29	5.49	-1.873202499

	AT5G59220	HAI1	72.94	117.53	119.93	2.29	10.27	10.72	-3.686652032
	AT1G07430	HAI2	40.24	29.1	36.89	11.88	12.58	7.13	-1.795211061
	AT2G23030	SNRK2.9	0.9	0.18	0.67	0.06	0.05	0	-4.179661095
	AT1G78290	SNRK2-8	12.67	14.85	12.76	25.86	59.98	25.66	1.474424541
ABF	AT2G41070	EEL	2.06	2.18	1.15	4.5	4.68	3.91	1.296699015
	AT2G36270	ABI5	9.33	11.34	10.63	7.05	3.92	3.43	-1.181149773
	AT4G35900	FD	3.73	1.76	1.6	20.21	7.57	17.47	2.701488457
ETR	AT1G04310	ERS2	3.9	7.6	4.09	1.38	1.49	2.26	-1.614882244
	AT3G23150	ETR2	8.6	13.04	6.81	1.92	3.38	2.94	-1.792788255
ERF1	AT3G23240	ERF1	61.61	51.99	53.17	15	14.99	43.12	-1.198012958

Table S3 DEGs involved in the Cysteine and methionine metabolism pathways

KEGG	Gene ID	Gene name	CdWT_1	CdWT_2	CdWT_3	CdOE5_1	CdOE5_2	CdOE5_3	log2FC
TAT;CORI3	AT4G23590		4.24	0.16	0.76	0	0.04	0.04	-5.975930434
serC, PSAT1	AT4G35630	PSAT	263.06	207.1	409.34	75.2	50.77	46.25	-2.414461417
	AT2G17630	PSAT2	7.73	5.35	7.36	2.31	0.75	1.45	-2.262985424
serA, PHGDH	AT1G17745	PGDH	515.08	754.42	579.04	302.34	112.95	185.34	-1.688033008
	AT1G17744		8.61	10.4	19.1	1.16	1.52	5.05	-2.27391228
	AT4G34200	EDA9	334.61	539.1	322.7	153.79	37.43	235.03	-1.510502997
cysK	AT5G28030	DES1	4.53	7.03	4.59	27.23	42.28	28.44	2.459748407
cysE	AT1G55920	SERAT2;1	113.28	187.15	175.1	34.79	56.66	56.63	-1.700001503
GOT1	AT5G19550	ASP2	133.84	168.1	168.22	90.56	70.66	78.62	-1.016302086
	AT1G62800	ASP4	0.86	1.57	0.88	8.42	9.88	4.99	1.995933215
	AT5G11520	ASP3	198.49	272.26	392.14	45.36	55.23	59.54	-2.456526022
MDH1	AT5G56720	c-NAD-MDH3	1.3	1.28	0.52	0.23	0	0	-4.108832656
	AT3G15020	mMDH2	37.27	38.82	41.82	14.89	11.86	12.95	-1.165503237
	AT5G09660	PMDH2	444.1	219.8	270.66	482.52	749.79	767	1.083891524
E2.6.1.42, ilvE	AT3G49680	BCAT3	26.61	22.2	21.58	61.77	51.26	73.22	1.346633355
AGXT2	AT3G08860	PYD4	78.29	147.78	127.46	9.67	17.34	5.47	-3.15166848
gshA	AT4G23100	GSH1	339.01	572.57	482.56	248.13	154.15	272.72	-1.055233828
thrA	AT1G31230	AK-HSDH I	13.03	11.72	9.07	40.6	27.61	33.86	1.563106282
metK, MAT	AT2G36880	MAT3	265.35	169.71	241.1	127.86	92.13	67.53	-1.294021628
speD, AMD1	AT3G25570	SAMDC3	13.23	6.24	8.27	38.3	36.85	48.65	2.137530693
ACS1_2_6	AT1G01480	ACS2	18.38	35.83	33.9	1.99	1.07	5.09	-3.519341992
ACS	AT2G22810	ACS4	0	0	0	0.24	0.42	0.31	5.628882522
	AT4G26200	ACS7	1.01	1.45	3.04	0.39	0.33	0.19	-2.662325671
E4.4.1.11	AT1G64660	MGL	248.83	158.05	163.73	22.51	12.81	18.57	-3.453374553

TAT	AT5G53970	TAT7	57.36	80.09	110.76	18.63	43.69	23.3	-1.551789019
	AT4G23590		4.24	0.16	0.76	0	0.04	0.04	-5.975930434
	AT2G24850	TAT3	412.26	332.25	455.8	91.05	78.72	81.62	-2.234670167
E1.14.17.4	AT1G62380	ACO2	866.55	1358.79	1581.7	237.46	184.4	252.85	-2.540270048
	AT1G12010	ACO3	3.05	1.11	2.14	4.79	9.79	10.2	1.978310427
mtnD, mtnZ, ADI1	AT2G26400	ARD3	1.14	3.61	29.41	3.02	1.16	1.28	-2.656571751

Table S4 Down-regulated genes related to lignin metabolic process

Gene id	Gene name	CdWT_1	CdWT_2	CdWT_3	CdOE5_1	CdOE5_2	CdOE5_3	log2FC
AT4G34050	CCoAOMT1	446.97	358.81	642.77	227.01	297.9	163.67	-1.115830897
AT1G61820	BGLU46	10.05	18.5	15.95	1.2	1.2	3.58	-2.994293577
AT5G13420	TRA2	82.72	91.27	110.15	39.14	29.97	21.98	-1.702293087
AT2G36880	MAT3	265.35	169.71	241.1	127.86	92.13	67.53	-1.294021628
AT5G60020	LAC17	5.59	2.06	1.26	0.66	0.06	0.08	-3.653178114
AT2G40890	CYP98A3	59.13	79.82	60.3	30.65	23.97	37.52	-1.143821171
AT5G48930	HCT	116.95	170.76	136.25	59.88	46.18	52.61	-1.462939163
AT1G79180	MYB63	1.57	1.43	1.9	0.23	0.11	0.17	-3.469348517
AT4G36220	FAH1	211.39	289.26	331.56	157.73	131.77	83.03	-1.218368935
AT4G37980	ELI3-1	457.95	502.07	439.63	142.49	129.22	156.76	-1.744835107
AT5G03260	LAC11	3.97	7.26	1.42	0.12	0.06	0	-6.275260475
AT4G37990	ELI3-2	247.92	289.95	360.09	27.38	26.98	32.11	-3.396213425
AT1G72680	CAD1	96.78	122.99	197.2	20.22	39.42	24.74	-2.319898722
AT5G05340	PRX52	26.5	72.69	46.6	12.75	12.41	9.04	-2.148370566
AT2G38080	IRX12	4.28	4.17	1.3	0.52	0.31	0.25	-3.230658934
AT4G34230	CAD5	230.73	276.35	378	74.7	105.22	64.37	-1.892085301
AT1G67980	CCOAMT	92.76	124.45	152.8	25.4	66.39	29.2	-1.605411474
AT5G54160	OMT1	203.06	208.57	271.53	91.3	117.58	58.62	-1.393608119
AT1G61810	BGLU45	15.31	16.3	17.2	0.4	1.5	4.38	-3.093789424
AT4G01070	GT72B1	49.52	75.76	79.63	53.27	31.73	19.77	-1.045743342
